# B2M mutation paves the way for immune tolerance in pathogenesis of Epstein-Barr virus positive diffuse large B-cell lymphomas

**DOI:** 10.7150/jca.75813

**Published:** 2022-11-07

**Authors:** Miaoxia He, Bin Liu, Gusheng Tang, Lijuan Jiao, Xuefei Liu, Shuyi Yin, Tao Wang, Jie Chen, Lei Gao, Xiong Ni, Libin Wang, Lili Xu, Jianmin Yang

**Affiliations:** 1 Department of Pathology, Changhai Hospital, Second Military Medical University, Shanghai, China 200433.; 2 Departments of Hematology, Changhai Hospital, Second Military Medical University, Shanghai, China 200433.

**Keywords:** Epstein-Barr virus, EBV+DLBCL, Gene mutation, B2M, Immune tolerance.

## Abstract

This study focused genetic pathogenesis and tumor microenvironment of Epstein-Barr virus (EBV) positive diffuse large B-cell lymphomas (DLBCL) in patients without immunodeficiency. DNA samples from these cases were sequenced by next generation sequencing (NGS) using a selected gene panel. Results revealed that most gene mutations were not specific for EBV positive DLBCL. However, B2M (β2-microglobulin) mutations were significantly increased and HLA-I or HLA-II expression was decreased in these cases, which was related to patient's poor outcome. B2M mutations and deregulation of B2M expression were further confirmed by Sanger sequencing and immunohistochemistry. Reducing the infiltration of CD8+ T lymphocytes, related to decreased expression of HLA-I or HLA-II was found in these patients. These results suggest that the mutations of B2M could cause the disruption of the expression and functions of this important subunit of HLA, leading to decreased expression of HLA-I or HLA-II and subsequently to reduce T lymphocyte infiltration in tumor tissues. The consequence of this event lessens the recognition and elimination of EBV+ tumor cells by host immunity and paves the way for the host immune tolerance to EBV+ tumor cells by evading immune recognition and escaping the T lymphocyte killing.

## Introduction

Epstein-Barr virus (EBV) positive diffuse large B-cell lymphoma is a proposed entity as a subtype of DLBCL without age limitation in the revised World Health Organization (WHO) classification of lymphoid neoplasm. 1-4. It accounts for 3% to 15% of DLBCL cases reported from different countries in different age groups^1^,^2^,^5^,^6^. Although the importance of the EBV positive DLBCL has been well established, data regarding outcome of this tumor are conflicting as to its special etiology and pathogenesis^7^-^9^.

EBV is typically transmitted in early human life as a subclinical illness^10^. Most humans tolerate latent EBV infection without adverse effects^10^. EBV is causally associated with some malignancies in certain individuals^11^. By regulating different pathways, EBV promotes survival of B cells and is implicated in the pathogenesis of DLBCL^5,6,12,13.^ EBV intrinsic mechanisms and human immunoreaction with this virus maybe play essential roles in EBV positive DLBCL development^10^-^13^. The etiology might be possibly associated with the immune tolerance induced by EB virus, but the mechanism was far from clear until now^7^-^9^.

The tumor microenvironment (TME) and limited immune surveillance play important roles in the lymphoma pathogenesis and clinical outcome^13^,^14^. In EBV positive DLBCLs, this microenvironment is possibly essential for tumor cells to interact with a variety of non-malignant cells in the EBV positive milieu and to create a special immunosuppressive microenvironment^15^-^16^. The immune escape from important immune cells such as T-lymphocytes and macrophages leads to the evasion of immune recognition^17^. However, the connection of the immune microenvironment to the pathogenesis and tumor survival of EBV positive DLBCL is still poorly understood^14^,^18^,^19^.

The aim of present study was to investigate the tumor microenvironment and molecular mechanisms of the immune tolerate in EBV positive DLBCLs.

## Material and methods

### Case selection

Pathology database of Department of Pathology, Changhai Hospital (Shanghai, China) was searched for patients with diffuse large B-cell lymphoma. Three hundred forty-six cases were identified over a 4-years period (2013.01-2016.12). Nearly all patients were residing in the Eastern China. Patients with impairment of the immune system secondary to primary immunodeficiency, HIV infection, transplantation and autoimmune disease, or with previous solid malignant tumor were excluded. Other exclusion criteria were evidences of acute or recent EBV infection and specific lymphoma subtypes known to be associated with EBV, such as Burkitt's lymphoma, classical Hodgkin lymphoma, lymphomatoid granulomatosis, primary effusion lymphoma and plasmablastic lymphoma. The clinical and laboratory data were collected in the hospital patient data system, including disease location, B symptoms, lactate dehydrogenase (LDH), β2-microglobulin (B2M), peripheral blood counts, EBV serology, clinical stage, primary treatment, relapse, secondary treatment, current status, and date of last follow-up. Three pathologists reviewed all cases and a consensus was reached in terms of morphological subtypes according to the updated World Health Organization (WHO) classification of tumors of hematopoietic and lymphoid tissue^1^-^2^. This study was approved by the Institutional Review Board of Changhai Hospital. All patients were provided with a written consent of sample collection for research use. The study was performed in accordance with the Declaration of Helsinki and was approved by the local ethics review committee.

### Immunohistochemistry and *in situ* hybridization studies

Formalin-fixed paraffin-embedded (FFPE) tissue microarray (TMA) was prepared from 180 cases and utilized for the immunohistochemical (IHC) and *in situ* hybridization analyses. The remaining 166 cases were performed on 3 µm FFPE tissue sections. Besides the routine diagnostic panel, IHC antibodies used in this study also included CD3, CD4, CD8, CD68, CD163, B2M, PD1, PDL1, EBNA2, LMP1, HLA-I (HLA-ABC), HLA-II (HLA-DR) and Ki-67. Particularly, the mismatch repair (MMR) markers containing MLH1, MSH2, MSH6 and PMS2 were detected. Immunohistochemistry were performed by EnVision Plus two-step system on an auto-Immunostainer (Bond, Leica, Germany). *In situ* hybridization for Epstein-Barr virus (EBV)-encoded small RNAs (EBERs) was conducted on formalin-fixed, paraffin-embedded sections with an FITC-labeled oligonucleotide probe supplied by Ventana on an automated stainer (Ventana Benchmark, Tuscon, AZ, USA). Positive and negative controls were ran along with all cases. The negative control sections were incubated with pre-immune serum. Three representative microscopic fields from each case were photographed with a 20×objective lens that covered a photographic area of 0.57 mm^2^. EBERs positivity was scored when positive neoplastic cells reached the 50% threshold. Immunohistochemistry results were evaluated by Allred score^20^.

### Genomic DNA Extraction and Next generation Sequencing

Genomic DNA was extracted from tumor tissues of EBV positive and negative cases. Targeted next generation sequencing (NGS) was conducted^21^ using a 82 genes panel (1914 coding exons). Among them, at least 11 genes (*PTEN, PIK3CA, ID3, PTPN6, B2M, CDX2NA, KMT2D, TCF3, MYD88, MYC, TP53*) are associated EBV associated malignancies including nasopharyngeal carcinoma, gastric cancer, Hodgkin's lymphomas and other lymphomas^23^-^24^. We performed gene mutation analysis on highly covered samples sequenced on the Illumina Hiseq1500TM system. The coverage of every target region of the samples was internally normalized, compared to normalized control data with other samples in the same run.

### Verification by Sanger sequence

In order to verify specific and interesting gene mutations obtained from NGS, the PCR products were purified using Exo SAP-IT (Affymetrix/USB, Santa Clara, Calif) and Qiaquick PCR Purification Kit (Qiagen, Valencia, CA). The purified samples were directly sequenced from both directions on the Applied Biosystems3130 Genetic Analyzer (Thermo Fisher Scientific Inc). Negative controls with wild-type genes were included in Sanger sequencing. As controls, the Burkitt's lymphoma cell line DAUDI and DLBCL cell line LY10 were also used for detecting B2M mutation.

### Statistical methods

Gene mutations and verification data were analyzed along with the immunohistochemical results and patients' follow-up information. Overall survival (OS) was defined from the date of the diagnosis until the date of death or last follow-up. Survival distributions were estimated with the Kaplan-Meier method and significance of difference between pairs was determined by the log-rank test. Analyses were performed using SPSS23 (SPSS Institute, Inc, Chicago, US). All P values are 2-tailed and presented without any adjustment for multiple comparisons. P<0.05 was considered significant.

## Results

### Clinical and Morphological Characteristics

The current study included 346 patients with DLBCL diagnosed in Changhai Hospital. All specimens were obtained at the time of the initial presentation. Clinical data were summarized in Table 1.

All patients presented with lymphadenopathy that was either localized (n=52) in a single location or at multiple sites (n=111). Mediastinal disease was present in 26 patients (7.6%). Extranodal non-lymphoid organ involvement was observed in 175 patients (50.5%), which included bone (n=26), liver (n=15), and lung (n=7). Clinically, 8% of patients (28/346) presented with splenomegaly, 47.4% had B symptoms, 28.9% had increased LDH, and 34.1% had elevated serologic B2M. Morphologically, there were two main histological patterns according to the WHO classification, which included: DLBCL-NOS (n=344) and THRLBCL-like (n=2). Immunohistochemically, tumor cells had an intact B-cell phenotype with the expression of CD20, CD79a and PAX5. All 346 cases were analyzed for CD10, Bcl-6 and MUM1 expression in order to evaluate their B cell origin. About 51.7% of the cases were GCB (germinal-center B-cell-like) subtype and the remaining cases were non-GCB subtype. Treatment and follow-up information were obtained for 346 patients (100%). The clinical stage was known for 346 cases: 52 were at stage I, 56 stage II, 93 stage III, and 145 stage IV. 68.8% of patients presented at advanced stage of the disease. All patients were treated with the combination of rituximab and other drugs. No patient received rituximab alone. The most common regimen used was rituximab, cyclophosphamide, doxorubicin, vincristine, and prednisone (346/346, 100%). Local radiation therapy was also applied to 37 patients (10.7%) (Table 1).

### EBV detection and following-up

**Figure 1:**
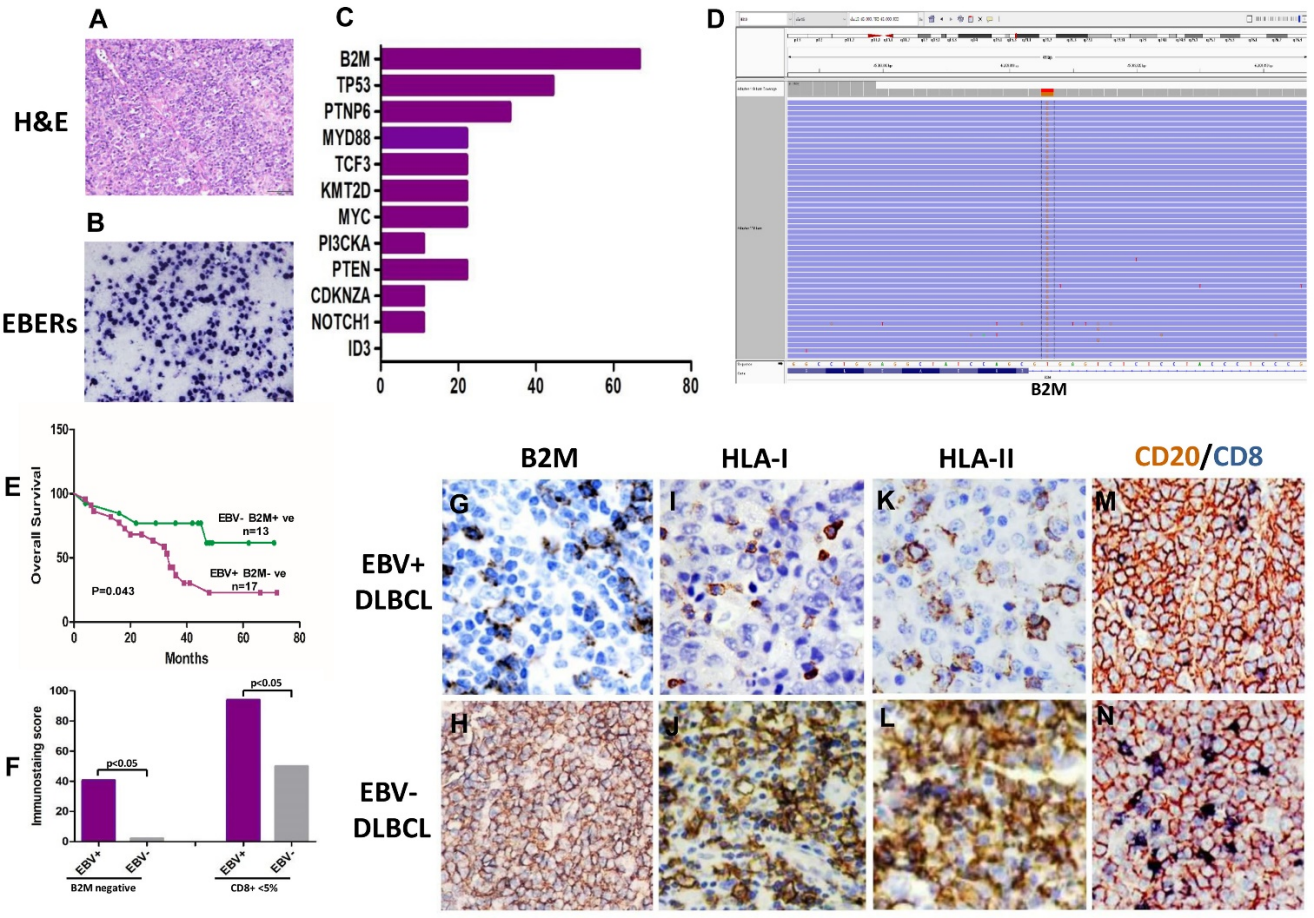
** B2M mutation, the expression of B2M, HLA class I and II and cytotoxic T-cell infiltration frequent in EBV positive DLBCLs. (A)** Morphology of EBV positive DLBCL showed typical morphology of DLBCL-NOS (H&E×400). **(B)** Tumor cells were positive for EBERs *in situ* hybridization (ISH×400). **(C)** The percentage of gene mutations identified by NGS in EBV positive DLBCLs. **(D)** A representative - B2M mutation (exon1, c.67+2T>G) in EBV positive DLBCL tissue. **(E)** The overall survival of EBV positive DLBCL patients with B2M inactivation is shorter than those with EBV negative DLBCL and no B2M mutation (p=0.043**). (F)** A comparison of the CD8 + infiltrated T Lymphocyte immunostaining score in tumor tissues between EBV positive DLBCL and negative ones (p<0.05) with and without B2M mutation. The data show that the immunostaining score in EBV+ DLBCLs was significantly higher than that in EBV- ones. B2M mutation(s) further increased the immunostaining score in both types of DLBCL tissues. **(G-L)** Representative images of the expression of B2M, HLA-I and HLA-II in DLBCLs. The expression of these three molecules in EBV positive DLBCLs detected by IHC was much less than that in negative ones (IHC×400). M &N. Double immunostaining revealed that CD8 + infiltrated T Lymphocytes (blue stained) in EBV positive DLBCL tumor tissues were less than in negative ones (IHC×400), while CD20 staining as a control was similar between EBV positive and negative DLBCL tumor tissues.

We observed the EBV infection status and clinico-pathological features of 346 DLBCLs cases of different ages. One of the important criteria for the diagnosis EBV positive DLBCL is that enrolled patients must be lack of history of immunodeficiency or immune compromise. The evidence of EBV infection is that more than 50% of the viable lymphoma cells are positive for EBERs by ISH. By this criterion, the positive for EBERs was found 10.7% (36/346) including DLBCL-NOS (n=34) (Fig 1A, 1B) and THRLBCL-like LBCL (n=2) cases. LMP1 was present in 18/346 (5.3%) of all analyzed cases. EBNA2 was negative in 34/36 (94%) cases. These results were consistent with EBV latency type II. EBV serologic data were available in 24/36 patients. All showed evidences of past infection: EBV viral capsid antigen IgG positive and EBV viral capsid antigen IgM negative. Twenty cases tested were also EBNA1 IgG positive. Among these 24 patients, 15 were positive for EBV early antigen IgG which was an indicator of virus reactivation.

The common sites of EBV positive DLBCL were cervical (n=11), axillary (n=4), and supraclavicular (n=3). Five out of 36 EBV positive DLBCL cases (13.9%) had bone marrow involvement. For the treatment, only 2 patients received high-dose of carmustine, etoposide and cytarabine, as well as autologous stem cell transplantation (ASCT). Overall, with a median follow-up of 34 months (range 1-83 months), 66.7% (24/36) patients were alive with no evidence of the disease, 3.2% (1/36) were alive with lymphoma, and 30.3% (11/36) died of the disease. Surprisingly, there were no significant clinical differences between EBV positive DLBCLs and EBV negative DLBCL cohort in terms of age, gender, subtype, revised International Prognostic Index (R-IPI), Eastern Cooperative Oncology Group (ECOG) score, Ann Arbor stage, B symptoms, bone marrow involvement, serologic LDH and B2M, chemotherapeutic regimen and double/triple gene alteration (C-MYC, BCL2 and/or BCL6) detected by FISH and/IHC. EBV detection and clinical data are summarized in Table 1.

### EBV infection and Gene mutation

For further understanding the genetic changes of EBV positive DLBCL, nine cases with more than 80% EBV positive cells in tumor tissue were selected for detecting gene mutation by NGS. For the comparison, nine cases of EBV negative DLBCL were also evaluated by NGS at the same time. NGS results from EBV positive DLBCLs revealed the presence of recurrent alterations in B2M (6/9; 66.7%) and PTPN6 (3/9; 33.3%), which were related to the tumor microenvironment and JAK-STAT pathways, respectively. The other mutated genes in EBV positive DLBCL were including MYC (2/9; 22.2%), TP53 (3/9; 22.2%), KMT2D (2/9; 22.2%), MYD88 (2/9; 22.2%), PIK3CA (2/9; 22.2%) NOTCH 1 (2/9; 22.2%) and TCF3 (1/9; 11.1%). Mutations found in EBV negative DLBCLs included TP53 (3/9; 22.2%), TCF3 (2/9; 22.2%), CDKN2A (3/9; 33.3%), MYC (2/9; 22.2%), ID3(2/9; 22.2%), PTEN (2/9; 22.2%) and B2M (1/9; 11.1%). The results showed no specific mutations on PTEN, ID3, CDKN2A and NOTCH1 genes in EBV positive DLBCLs. Though PTPN6 and TP53 were found to be mutated in different exons with high relative percentage, the results did not reach statistical significance between EBV positive and negative DLBCLs. B2M was only gene to stand out regarding mutations among the most high-frequently mutated genes between these two groups. B2M mutation was found 66.7% (6/9) in EBV positive cases, while EBV negative cases were 11.1% (1/9) (Fig 1C, 1D).

### B2M mutation verification

Because B2M gene is an invariant subunit of the class I human leukocyte antigen complex (HLA-I) involved in the process of presenting antigenic peptides derived from degraded endogenous self- or non-self-proteins, including viral- or tumor- associated antigens^14^,^25^,^26^. From our data, we noticed B2M mutation rate is higher in EBV positive DLBCLs than negative cases. Then, we verified the mutations of B2M coding exons in all EBV positive DLBCL cases and another 20 cases of EBV negative DLBCL using Singer sequencing. Results showed that missense mutations (n=14) affected the start codon for methionine and converted it to arginine, lysine, or threonine (ATG to AGG/AAG/ACG) 27. Special point mutations (exon1: c.67+2T>G, n=3) are expected to inactivate protein function of B2M based on the PolyPhen prediction algorithm, resulting in transcripts that encode truncated B2M proteins. The overall mutation of B2M is about 41.7% (15/36) in the EBV positive DLBCL cases. The analysis of paired EBV negative DLBCL cases indicate that the overall B2M mutations including nonsense mutations and some somatic events, accounted for 2.1% (n=6/29) of the EBV negative DLBCL samples analyzed. The control Burkitt's lymphoma cell line DAUDI was found having the same missense mutation ATG to AGG as previously documented 27, while the DLBCL cell line LY10 was negative for B2M gene mutation.

### Analyzing B2M expression and T cell infiltration

The majority of the B2M mutations identified were represented by unambiguously inactivating events with immune abnormality^14^,^28^. The association of B2M gene alteration with defective HLA-1 expression was reported in several cancers, including colorectal carcinoma and lymphomas. Furthermore, the B2M expression has been studied by immunohistochemistry in both EBV positive and negative cases. The lack of cell surface HLA-1expression due to different mechanisms such as DNA mismatch repair (MMR) allows tumor cells to escape from immune recognition by T lymphocytes in colorectal carcinoma^29^. Therefore, we particularly analyzed the expression of B2M, HLA-I, HLA-II, PD1, PDL1, M2 macrophages infiltration, MLH1, MSH2, MSH6 and PMS2 for MMR status in both EBV positive and negative lymphoma cases. We used the double staining of CD20 and CD8 or CD4 to highlight infiltrated T-lymphocyte in tumor tissues.

The expression of B2M was down regulated. The positive rate in EBV positive DLBCL cases was 47.2% (17/36). The expression of HLA-I or HLA-II were also significantly decreased in the B2M expression-impaired cases (13/17, 76.4%). Immunohistochemical staining results revealed decreased infiltration of cytotoxic T cell (< 5%) in most of these cases (16/17). The B2M mutations and decrease in cytotoxic T cells in EBV positive DLBCL were correlated with patient's poor prognosis compared to EBV negative and B2M normally expressed cases (p=0.043) (Fig 1E,1F). Among EBV negative DLBCLs, only a few cases showed HLA-I or HLA-II negative (n=2), and decreased infiltration of CD8+ T cell (< 5%, n=3). Interestingly, the serum level of B2M did not show the correlation with the cases of B2M negative in tumors, and it did not reach significant difference between EBV positive and negative group either. On the other hand, the percentage of both CD20-/CD4+ T cells and CD20-/CD8+ T cells in EBV positive DLBCLs were much less than those in EBV negative cases. Additionally, the ratio of CD4: CD8 T cells was higher in EBV positive cases than EBV negative ones, which was related to the less infiltration of CD8+T lymphocytes (Fig 1G-1N) in EBV+ cases. In this study, the M2 macrophages (CD163+ and CD68+) displayed the same infiltration pattern in EBV positive and negative cases, though the percentage of M2 macrophages in EBV positive cases was slightly more than that ib negative cases (15/36 Vs 12/29, p>0.05). Meanwhile, we found the deficiency of MMR (dMMR) in one EBV positive DLBCL and two EBV negative DLCBL cases. These cases also had MSH2 and MSH6 deletions^29^. In addition, two group didn't show significant difference in the expression of PD1 and PDL1 (8 /36 Vs 7/29; 10 /36 Vs 8/29).

**Figure 2:**
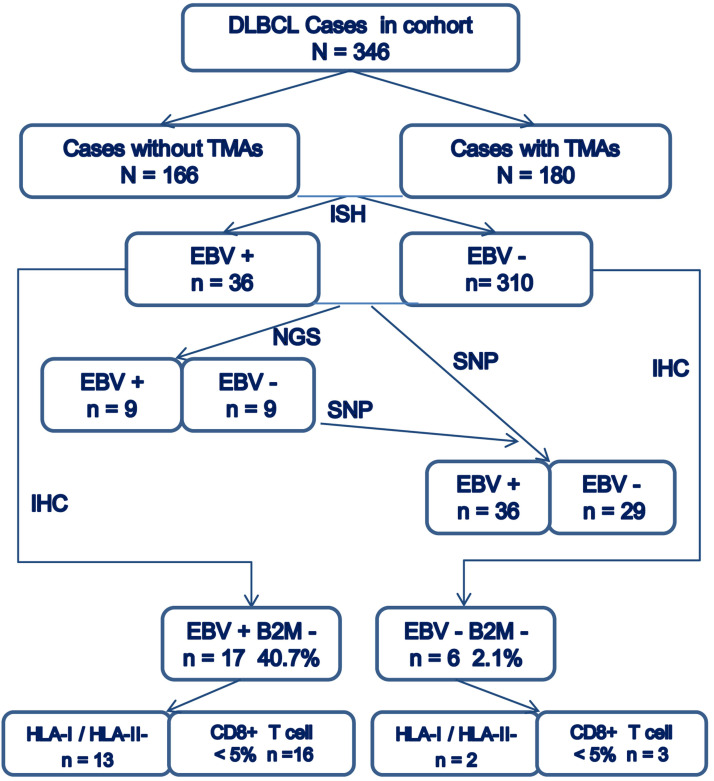
Consort diagram of cases included in the present study.

The overall consort diagram of cases included in this study was shown in Figure 2. The multivariable statistics showed that patient's age, B2M mutation and expression, HLA-I or HLA-II decreased expression, and infiltration of CD8+T lymphocytes were significantly associated with patient survival (Table 2).

## Discussion

EBV has been closely linked to various malignant tumors. However, the detail pathogenesis is still not very clear^10^,^11^. It is possible that the development of EBV associated tumors is related to virus induced gene abnormalities and tumor microenvironment^15^,^16^. EBV positive DLBCLs have been re-recognized in recent years because of controversial clinical outcomes from different areas^4^,^5^,^7^,^8^. Here, we present a systematic evaluation of EBV status and clinicopathological features of 346 DLBCL patients resided in Eastern China. EBERs were found 10.7% of all tested cases and 94% of them was EBV latency II (EBNA2 negative) without immunodeficiency. In order to better understand this type of special EBV related tumor, we evaluated gene mutations by NGS in nine cases of EBV positive DLBCL, which contained more than 80% EBERs positive cells in their tumor tissues. The panel of genes was specifically selected from literature about EBV associated tumors including nasopharyngeal carcinoma, gastric cancer, Hodgkin's lymphomas and other lymphomas^21^-^23^. Unfortunately, the results showed that there was no difference in specific gene mutations between EBV positive DLBCLs and other EBV associated tumors^21^-^23^. Although PTPN6 and TP53 were found mutations in different hot exons with high relative percentage, the results did not reach statistical significance between EBV positive DLBCL and negative ones. However, we noticed that B2M mutation was outstanding among other gene mutations. Its rate was significantly higher in EBV positive cases (66.7%) than in EBV negative cases (11.1%). Further verification of the B2M mutations by Sanger sequencing confirmed B2M mutations status (40.7%) in EBV positive cases. B2M protein expression detected by Immunohistochemistry revealed that only 2.7% of these positive cases had normal B2M expression, significantly lower than in B2M un-mutated cases, EBV negative cases. B2M gene alteration and decreased expression were correlated with patient poor prognosis, compared to EBV negative cases (p=0.043). The data present a special finding in EBV positive DLBCL patients, comparing with those reported in previous literature^4^-^7^.

Tumor microenvironment is very intriguing, in that it plays a critical role in the regulation of tumor cell survival and proliferation as well as fostering immune escape^15^,^16^,^24^. B2M gene encodes the protein B2-microglobulin that is essential for MHC class I complex formation and peptide function^25^-^26^. The MHC-I molecule is composed of HLA -I and B2M protein that acts as a stabilizing scaffold. Sense mutations and deletions in the B2M gene could crucially impair MHC-I assembly and cell surface expression^27^-^28^. In particular, B2M gene alterations associated with defective HLA-I expression have been reported in a small number of lymphoma cases^29^. Furthermore, we found that the expression of HLA-1 or HLA-II was much severe down regulated in EBV positive DLBCL than negative ones that also had B2M mutation(s). Miyashita K. and his colleagues previously also reported that B2M mutations were linked to decreased MHC -1 expression and decreased patient survival in DLBCLs^30^. Because B2M is involved in the presentation of antigenic peptides derived from degraded endogenous self- or non-self-proteins, including viral- or tumor-associated antigens^24^-^28^. Therefore, we believe that in EBV infected DLBCL, B2M mutations could cause to HLA-I and/or HLA-II associated abnormal immune tolerance to EBV virus and viral infected cells, leading to the development and progress of EBV infected DLBCL.

T lymphocytes play a major role in cell-mediated immunity. As part of HLA molecules, B2M is essential for MHC-I complex formation and peptide presentation^25^-^27^. The MHC-I molecules display intracellular peptides on the cell surface for the “inspection” by CD8+ T cells^31^-^33^. These T cells have the potential to recognize the MHC-I peptide complex and become activated cytotoxic T cells. In general, immune evasion is a patho-genetic mechanism used by several types of cancers in their evolution and escape from CD8 + T cells recognition. Also, one of the main functions of CD4+ T cells is the regulation of antitumor immune responses by suppressing the proliferation of CD8+ T cells, which may lead to an immune escape and to the proliferation of cancer cells. Additionally, we used double staining of CD20 and CD8 or CD4 highlighting the infiltrated T-lymphocyte in tumor tissues. Our results indicated that both CD20-/CD4+ T cells and CD20-/CD8+ T cells infiltration in EBV positive DLBCLs were fewer than those in EBV negative cases, while the ratio of CD4:CD8 T cells in EBV positive DLBCLs was higher than the ratio in EBV negative cases. This is possibly related to decreased infiltration of CD8 + T cells within lymphoma tissues in EBV positive DLBCL cases. Meanwhile, in a last extensive study, it was found that the loss of HLA class I and II expression on DLBCL cells was associated with low tumor infiltration by CD8+ T cells 32. We recognized that reduced membranous staining of HLA-I and HLA-II molecules in EBV positive B2M mutated DLBCLs was correlated with low level of CD8+ T-cell infiltration. These results suggest that defects in HLA complexes due to B2M inactivate might impair the recruitment of the tumor-infiltrating CD8+ T-lymphocytes in EBV positive DLBCLs, which induces host immunotolerance to viral infection and tumor cells and leads to tumor development and progress.

B2M gene alterations have been reported to associate with defective HLA-1 expression in colorectal carcinoma. Through heterogeneous mechanisms such as MMR, tumor cells could escape from immune recognition by T lymphocytes^32^. We particularly analyzed the MMR and T lymphocyte infiltration in both EBV positive and negative cases too. We found dMMR in one case of EBV positive DLBCL and in two cases of the EBV negative ones. All of them showed MSH2 and MSH6 deletion without correlation to T lymphocyte infiltration. NK cell and macrophages are also believed to be very important in tumor microenvironment^15^,^16^,^32^. However, we found that there was no significant difference in the number of infiltrated CD56+ NK cells, CD163 and CD68+ M2 macrophages between EBV positive and negative groups except slight M2 macrophages polarity in EBV positive cases(p>0.05). The expression of PD1 and PDL1 between two groups did not show significant difference in our cases^34^,^35^. Therefore, B2M gene dysfunction played a crucial role in impairing MHC class I assembly and HLA cell surface expression, which helps tumor cells escape from the recognition by cytotoxic T cells^32^,^36^. This cascade event is closely related to HLA -I expression and CD8+ T lymphocytes infiltration, and partially HLA -II expression^37^, which induce immune toleration. However, there was not definite correlation to macrophages infiltration in tumor microenvironments of EBV positive DLBCL. Though B2M mutation is closed associated with dMMR in colorectal carcinoma^33^, we did not get significant mismatch repair events in EBV related DLBCLs. We also noticed that there was no relationship between B2M mutation and the expression with serologic B2M level in EBV positive DLBCLs, which could be due to the secretion of large amount of B2M protein by the vast number of unaffected cells in the body.

In summary, EBV represents a foreign antigen against host cytotoxic T lymphocyte immunity^15^,^16^,^32^. EBV within the lymphoma cells is strongly implicated in mechanisms of immune evasion by complex mechanisms^9^,^12^,^13^. The present consort prompts us to believe that B2M mutations in EBV positive DLBCLs might pave the way for the host immunotolerance to tumor cells through the deregulation of HLA expression and impairment of CD8+ T lymphocyte infiltration. The consequence of this immunotolerance is to allow the tumor cells to evade human immunity and contribute the development and progress of tumors, leading to the poor outcome of DLBCL patients. The results presented herein are novel and emphasize the importance of B2M mutation as a mechanism regulating host immune toleration to EBV+ tumor cells which evade immune recognition and escape the T lymphocytes killing in EBV positive DLBCLs.

## Supplementary Material

Supplementary tables.

## Figures and Tables

**Table 1:** Clinical characteristics of 346 cases of DLBCL patients.

Parameters		EBERs		
	Total	Positive	Negative	*P* value
Overall	346(100%)	36	310	
Age				0.064
<50y	104(53.2%)	6(16.7%)	98(31.6%)	
≥50y	242(46.8%)	30(83.3%)	212(68.4%)	
Gender				0.145
Female	155(44.8%)	17(47.2%)	138(44.5%)	
Male	191(55.2%)	19(52.8%)	172(55.5%)	
Subtype *				0.419
GCB	178(51.7%)	15(44.1%)	163(52.9%)	
Non-GCB	166(48.3%)	19(55.9%)	145(47.1%)	
IPI score				0.987
0-1	133(38.5%)	14(38.9%)	119(38.4%)	
2-3	152(43.9%)	16(44.4%)	136(43.9%)	
4-5	61(17.6%)	6(16.7%)	55(17.7%)	
ECOG				0.222
0-1	242(69.9%)	22(61.1%)	220(71.0%)	
≥2	104(30.1%)	14(38.9%)	90(29.0%)	
Ann Arbor stage				0.928
I-II	108(31.2%)	11(30.6%)	97(31.3%)	
III-IV	238(68.8%)	25(69.4%)	213(68.7%)	
B symptoms				0.165
Yes	164(47.4%)	21(58.3%)	143(46.1%)	
No	182(52.6%)	15(41.7%)	167(53.9%)	
Bone marrow involvement				0.711
Yes	37(10.7%)	5(13.9%)	32(10.3%)	
No	309(89.3%)	31(86.1%)	278(89.7%)	
LDH				0.875
<310	246(71.1%)	26(72.2%)	220(71.0%)	
≥310	100(28.9%)	10(27.8%)	90(29.0%)	
β2-MG				0.312
<2.8	228(65.9%)	21(58.3%)	207(66.8%)	
≥2.8	118(34.1%)	15(41.7%)	103(33.2%)	
Chemotherapeutic regimen				0.662
RTX-containing	229(66.2%)	25(69.4%)	204(65.8%)	
RTX-absent	117(33.8%)	11(30.6%)	106(34.2%)	
Double expression				0.891
Yes	26(7.5%)	2(5.6%)	24(7.7%)	
No	320(92.5%)	34(94.4%)	286(92.3%)	

Abbreviations: DLBCL, diffuse large B-cell lymphoma; GCB, germinal center B-cell; GCB, germinal center B-cell; IPI, International Prognostic Index; ECOG, Eastern Cooperative Oncology Group; LDH, lactate dehydrogenase; β2-MG β2-microglobulin. * This subtype did not include 2 cases of THRBCL.

**Table 2:** Multivariate Cox regression survival analysis of multiplex data.

Variables	Hazard ratio (HR)	95% Confidence Interval (CI)	*P value*
Ages	3.375	1.162-7.886	0.024
EBERs +	1.421	0.765-3.636	0.275
B2M mutation	3.637	1.174-4.657	0.001
B2M expression	4.705	1.485-10.657	0.006
Serologic B2M	1.968	0.893-10.125	0.146
HLA-I or II -	4.105	2.203-8.967	0.017
CD4 +	3.872	1.715-12.149	0.095
CD8 +	5.612	1.956-14.312	0.002
PDL1 +	1.872	0.852-11.234	0.115
PD1 +	3.872	2.412-5.113	0.317
CD163 + M2	4.627	0.994-13.785	0.054
dMMR	0.159	0.632-1.763	0.569

**Abbreviations:** EBERs, Epstein-Barr virus -encoded small RNAs. B2M, β2-microglobulin. M2, microphage 2. HLA, human leukocyte antigen complex. dMMR, Deficiency of mismatch repair.
