# Supplementary material for: B2M mutation paves the way for immune tolerance in pathogenesis of Epstein-Barr virus positive diffuse large B-cell lymphomas
Source: J Cancer. 2022 Nov 7;13(15):3615–22. doi: 10.7150/jca.75813 (PMC9809314; doi:10.7150/jca.75813)
Supplement: Supplementary file 1 — Supplementary tables. [file jcav13p3615s1.pdf]

Suppl. Table 1 Antibodies used for immunohistochemistry

Antibody	Clone	Manufacturer	Catalogue#	Dilution
CD3	LN10	ZSBio. Beijing, China	ZM-0417	Not
CD4	UMAB64	ZSBio. Beijing, China	ZM-0418	Not
CD8	SP16	ZSBio. Beijing,China	ZA-0508	Not
CD68	KP1	ZSBio. Beijing, China	ZM-0060	Not
CD163	10D6	ZSBio. Beijing, China	ZM-0428	Not
B2M	3F9-2C2	Abcam. Cambridge, UK.	Ab54810	1:50
PD1	PDCD1/922	Abcam. Cambridge, UK.	Ab216037	1:50
PDL1	SP142	Abcam. Cambridge, UK.	ab228463	1:50
EBNA2	PE2	Abcam. Cambridge, UK.	Ab90543	1:50
LMP1	CS1-4	Abcam. Cambridge, UK.	Ab78113	1:50
HLA-I (HLA-ABC)	W6/32	Abcam. Cambridge, UK.	Ab22432	1:50
HLA-II (HLA-DR)	LN3	Abcam. Cambridge, UK.	Ab212444	1:50
MLH1	MMR-Panel	Abcam. Cambridge, UK.	Ab252190	1:50
PMS2	MMR-Panel	Abcam. Cambridge, UK.	Ab252190	1:50
MSH2	MMR-Panel	Abcam. Cambridge, UK.	Ab252190	1:50
MSH6	MMR-Panel	Abcam. Cambridge, UK.	Ab252190	1:50
Ki-67	SP6	Abcam. Cambridge, UK.	Ab16667	1:50

Suppl. Table 2 Genes in the NGS targeted panel (82 genes panel)*

ACTB	*CD79A*	*FOXO1*	*MYC*	*SLITRK3*
B2M	*CD79B*	*GNA13*	*MYD88*	*SOCS1*
BCL2	*CIITA*	*HIST1H1C*	*MYOM2*	*STAT3*
BCL6	*CREBBP*	*IGLL5*	*NRXN3*	*STAT6*
BTG1	*CXCR4*	*IRF4*	*P2RY8*	*TBL1XR1*
BTG2	*DDX3X*	*IRF8*	*PCLO*	*TMSB4X*
CARD11	*DTX1*	*KMT2D*	*PIM1*	*TNFAIP3*
CCND3	*EBF1*	*LRRN3*	*POSTN*	*TNFRSF14*
CD20	*EP300*	*LYN*	*PRDM1*	*TP53*
CD58	*EZH2*	*MEF2B*	*ROBO2*	*TRAF2*
CD70	*FAS*	*MPEG1*	*SGK1*	*UBE2A*
PTEN	*CDKN2B*	*PTPN6*	*CDKN2A*	*NOTCH1*
PIK3CA	*KMT2A*	*TCF3*	*ID3*	*NOTCH2*
PD1	*PDL1*	*FOXP1*	*CDKN1A*	*CDKN2A*
ATM	*ARIDA*	*CCND1*	*JAK1*	*JAK2*
RHOA	*TET2*	*IDH2*	*FAT1*	*BRAF*
ALK	*XPO1*			

- The panel was created by Yuanqi BioPharmaseutics, Shanghai, China.
